# Supplementary material for: A decoherence-free subspace in a charge quadrupole qubit
Source: Nat Commun. 2017 Jun 23;8:15923. doi: 10.1038/ncomms15923 (PMC5490009; doi:10.1038/ncomms15923)
Supplement: Supplementary Information [file ncomms15923-s1.pdf]

File name: Supplementary Information
Description: Supplementary notes and supplementary references.

File name: Peer review file
Description:

In these Supplementary Notes, we explore several issues related to the operation of a charge quadrupole qubit.

Supplementary Note 1: Estimated size of dipolar vs. quadrupolar detuning fluctuations.

The charge quadrupole (CQ) qubit is less susceptible to charge noise than a charge dipole (CD) qubit because in solid state devices the dipolar component of the charge noise, $\delta\epsilon_{\rm d}$, is typically much larger than the quadrupolar component, $\delta\epsilon_{\rm q}$. Here, we estimate the relative strengths of these two components based on experimental measurements of charge noise in semiconducting qubit devices, assuming that both types of electric field noise arise from the same remote charge fluctuators.

We begin by considering charge noise from remote charge traps in the semiconductor device[1–4]. As a simple model, we consider a charge trap with two possible states: occupied vs. empty. Compared to a dipole fluctuator, in which the charge toggles between two configurations, the monopole fluctuator can be considered a worst-case scenario because the monopole potential decays as $1/R$ while the dipole potential decays as $1/R^2$, where R is the dot-fluctuator separation. Following ref. 5, this monopole model can be used to estimate the characteristic separation R between the fluctuator and the quantum dot, based on charge noise measurements in a double-dot charge qubit. Experimental measurements of the dephasing of charge qubits[1–4] yield estimates for the standard deviation of the dipole detuning parameter, σ_ϵ, which range between roughly 3 and 8 µeV for double dots separated by 200 nm, leading to estimates for the dot-fluctuator separation of $R \sim$ 1.1-2.5 µm. (Note that a significantly smaller σ_ϵ was recently reported in ref. 6, which would correspond to a much larger value of R.)

With this information, we can estimate the ratio $\delta\epsilon_{\rm q}/\delta\epsilon_{\rm d}$. In a worst-case scenario, corresponding to the strongest quadrupolar fluctuations, the monopole fluctuator would be lined up along the same axis as the triple dot. Adopting a point-charge approximation for the fluctuator potential, $V(r) = e^2/4\pi\varepsilon r$, where e is the charge of the electron, ε is the dieletric constant, and r is distance from the point-charge, and assuming an interdot spacing $d \ll R$, equation (2) of the main text yields

$$\frac{\delta\epsilon_{\rm q}}{\delta\epsilon_{\rm d}} \simeq \frac{d}{R}. \tag{1}$$

Taking $d = 200$ nm, and $R \simeq$ 1-3 μm, we estimate that $\delta\epsilon_{\rm q}/\delta\epsilon_{\rm d} \simeq$ 0.07-0.2 for typical devices. In other words, for current devices, the quadrupolar detuning fluctuations should be ∼10 times weaker than dipolar detuning fluctuations. Moreover, new generations of quantum dots in heterostructures without modulation doping[7–10] have the potential to achieve much smaller d, which would further suppress $\delta\epsilon_{\rm q}/\delta\epsilon_{\rm d}$.

In summary, we have estimated the characteristic separation R between a double dot and a charge fluctuator, based on measurements of charge noise in quantum dot devices. Of course, fluctuators are randomly distributed in solid-state systems, and it is possible for a defect to be located much closer to the qubit than our estimate suggests. Such noisy environments have a negative impact on both CD and CQ qubits. Fortunately, the length scales d and R appear to be well separated, so that fluctuations in a given qubit are very likely to be dominated by dipolar detuning fluctuations rather than quadrupolar fluctuations. In fact, the scaling expression in equation (1) is one of the most appealing arguments for exploring CQ qubits, which couple primarily to gradient field fluctuations, because the dephasing effects of the quadrupolar fluctuations can always be suppressed by reducing the device size and shrinking the interdot distance. Indeed, quantum devices with dot separations of $d \simeq 50$ nm have recently been reported[11], corresponding to a further reduction in $\delta\epsilon_{\rm q}/\delta\epsilon_{\rm d}$ by a factor of 4 compared to the estimate given above.

Supplementary Note 2: Quantum dot variability.

The combined requirements of $\bar{\epsilon}_{\rm d} = 0$ and $t_{\rm A} = t_{\rm B} \equiv t/\sqrt{2}$ indicate that the CQ dot geometry should be highly symmetric. Other types of symmetric geometries have also been proposed for improving the operation of charge-based qubits in superconducting Cooper-pair boxes[12–14], as well as an exchange-only logical spin qubit[15,16]. To achieve such symmetry in a triple-dot qubit, we must assume that $t_{\rm A}$ and $t_{\rm B}$ are independently tunable.

In the main text, we assume that uniform electric field fluctuations, δE, couple to $\epsilon_{\rm d}$ but not to $\epsilon_{\rm q}$. However, this statement contains some hidden assumptions about the symmetries of a triple dot, which may not be valid when we account for dot variability. Here, we show that if the triple-dot symmetry is imperfect, uniform field fluctuations could induce effective quadrupolar fluctuations $\delta\epsilon_{\rm q}$ that potentially spoil the CQ noise protection, and we explain how to avoid this problem.

Quantum dots are confined in all three dimensions. The vertical confinement is typically very strong, so we can apply the usual subband approximation and treat the dot in two dimensions (2D)[17]. Let us begin with a 1D parabolic approximation for the lateral confinement potential in a single dot:

$$V_i(x) = \frac{m\omega_i^2}{2}(x - x_i)^2 + U_{0i}, \tag{2}$$

where $i = 1, 2, 3$ is the dot index, m is the effective mass, $\hbar\omega_i$ is the splitting between the simple harmonic energy levels, x_i is the center of the dot, and U_{0i} is the local potential. A more accurate description of $V_i(x)$ could in-

clude anharmonic terms, which would yield higher-order corrections to the results obtained here.

The parameters ω_i, x_i, and U_{0i} all depend on voltages applied to the top gates. We assume that the U_{0i} terms are adjusted to satisfy the requirement that $\bar{\epsilon}_\mathrm{d} = 0$, and henceforth ignore them. The dot positions x_i can also be controlled electrostatically by tuning the gate voltages near the dot. The parameter ω_i is the most difficult to adjust after device fabrication, because it is mainly determined by the fixed top-gate geometry, or other fixed features in the electrostatic landscape. Electrons in dots with different ω_i respond differently to δE, and can therefore potentially affect the symmetries of a CQ qubit. However, we now show that dot-to-dot variations in ω_i do not couple to δE fluctuations at linear order.

A uniform fluctuating field δE introduces a term of form $-ex\delta E$ in the energy. Adding this term to equation (2) and rearranging yields

$$V_i(x) = \frac{m\omega_i^2}{2}(x - x_i')^2 - ex_i\delta E - \frac{e^2\delta E^2}{2m\omega_i^2}, \qquad (3)$$

where $x_i' = x_i + (e/m\omega_i^2)\delta E$ represents the shifted center of the dot. Considering the first term on the right-hand side of equation (3), we note that the energy of a shifted harmonic oscillator does not depend on its position, x_i'. Dot-to-dot variations in this term therefore do not depend on δE, and can be compensated by adjusting the potentials U_{0i}. The leading order fluctuation term in equation (3) is therefore $-ex_i\delta E$, which does not depend on ω_i. The coupling between δE and ω_i only arises at higher order, in the third term of equation (3).

The term $-ex_i\delta E$ in equation (3) can be viewed as a fluctuating site potential δU_i. The CQ symmetric design strategy provides a mechanism for eliminating the leading order dipolar detuning fluctuations. However, from the definition of the quadrupolar detuning in equation (2) of the main text, we see that the quadrupolar detuning fluctuations are given by

$$\delta\epsilon_\mathrm{q} = \delta U_2 - \frac{\delta U_1 + \delta U_3}{2} = e\left(-x_2 + \frac{x_1 + x_3}{2}\right)\delta E. \quad (4)$$

In other words, *uniform* electric field fluctuations can also generate *quadrupolar* detuning fluctuations in an asymmetric triple dot. Fortunately, it is straightforward to suppress this effect by adjusting the dot separations to make them equal:

$$x_2 - x_1 = x_3 - x_2 = d_x. \qquad (5)$$

Repeating this analysis for the dot confinement along the y axis, we obtain the additional requirement that

$$y_2 - y_1 = y_3 - y_2 = d_y. \qquad (6)$$

Hence, the three dots must be equally spaced along a line. These new symmetry requirements are not oppressive, and can be achieved by simply including two top gates to fine-tune the x and y positions of one of the dots; such fine-tuning can even be accomplished via automated methods[18]. Moreover, small errors in the dot position, δx, are tolerable since they only increase the detuning by a linear factor, $\delta\epsilon_\mathrm{q} = (\delta x/d)\delta\epsilon_\mathrm{d}$, where we have expressed the uniform field fluctuations in terms of the dipolar detuning parameter.

Supplementary Note 3: Details on the $\tilde{X}_\pi$ pulse sequence.

We consider the specific pulse sequence $\tilde{X}_\pi \equiv Z_{2\pi}X_{3\pi}Z_{-2\pi}$. A more general set of three-step sequences is discussed in ref. 19. For the bare $Z_{2\pi}$ gate, we choose $\epsilon_\mathrm{q} = \epsilon_z > 0$, with the corresponding gate time $\tau_z = h/\epsilon_z$. For the $Z_{-2\pi}$ gate, we replace $\epsilon_z \to (-\epsilon_z)$, but keep the same gate time. For the $X_{3\pi}$ gate, we set $t = t_x \equiv \epsilon_z/2\pi$, with gate time $\tau_x = 3h/4t_x$.

SUPPLEMENTARY REFERENCES

1. Dial, O. E., Shulman, M. D., Harvey, S. P., Bluhm, H. Umansky, V. & Yacoby, A. Charge noise spectroscopy using coherent exchange oscillations in a singlet-triplet qubit. *Phys. Rev. Lett.* **110**, 146804 (2013).
2. Petersson, K. D., Petta, J. R., Lu, H. & Gossard, A. C. Quantum coherence in a one-electron semiconductor charge qubit. *Phys. Rev. Lett.* **105**, 246804 (2010).
3. Buizert, C., Koppens, F. H. L., Pioro-Ladrière, M., Tranitz, H.-P., Vink, I. T., Tarucha, S., Wegscheider, W. & Vandersypen, L. M. K. *In situ* reduction of charge noise in GaAs/Al$_x$Ga$_{1x}$As Schottky-gated devices. *Phys. Rev. Lett.* **101**, 226603 (2008).
4. Shi, Z., Simmons, C. B., Ward, D. R., Prance, J. R., Mohr, R. T., Koh, T. S., Gamble, J. K., Wu, X., Savage, D. E., Lagally, M. G., Friesen, M., Coppersmith, S. N. & Eriksson, M. A. Coherent quantum oscillations and echo measurements of a Si charge qubit. *Phys. Rev. B* **88**, 075416 (2013).
5. Gamble, J. K., Friesen, M., Coppersmith, S. N. & Hu, X. Two-electron dephasing in single Si and GaAs quantum dots. *Phys. Rev. B* **86**, 035302 (2012).
6. Mi, X., Cady, J. V., Zajac, D. M., Deelman, P. W. & Petta, J. R. Strong coupling of a single electron in silicon to a microwave photon. *Science* **355**, 156-158 (2017).
7. Wu, X., Ward, D. R., Prance, J. R., Gamble, J. K., Mohr, R. T., Shi, Z., Savage, D. E., Lagally, M. G., Friesen, M.,

Coppersmith, S. N. & Eriksson, M. A. Two-axis control of a singlet-triplet qubit with an integrated micromagnet. *Proc. Nat. Acad. Sci.* **111**, 11938-11942 (2014).

[8] Veldhorst, M., Hwang, J. C. C., Yang, C. H., Leenstra, A. W., de Ronde, B., Dehollain, J. P., Muhonen, J. T., Hudson, F. E., Itoh, K. M., Morello, A. & Dzurak, A. S. An addressable quantum dot qubit with fault-tolerant control-fidelity. *Nature Nano.* **9**, 981-985 (2014).

[9] Borselli, M. G., Eng, K., Ross, R. S., Hazard, T. M., Holabird, K. S., Huang, B., Kiselev, A. A., Deelman, P. W., Warren, L. D., Milosavljevic, I., Schmitz, A. E., Sokolich, M., Gyure, M. F. & Hunter, A. T. Undoped accumulation-mode Si/SiGe quantum dots. *Nanotechn.* **26**, 375202 (2015).

[10] Zajac, D. M., Hazard, T. M., Mi, X., Wang, K. & Petta, J. R. A reconfigurable gate architecture for Si/SiGe quantum dots. *Appl. Phys. Lett.* **106**, 223507 (2015).

[11] Veldhorst, M., Yang, C. H., Hwang, J. C. C., Huang, W., Dehollain, J. P., Muhonen, J. T., Simmons, S., Laucht, A., Hudson, F. E., Itoh, K. M., Morello, A. & Dzurak, A. S. A two-qubit logic gate in silicon. *Nature* **526**, 410-414 (2015).

[12] Zhou, X., Wulf, M., Zhou, Z., Guo, G. & Feldman, M. J. Dispersive manipulation of paired superconducting qubits. *Phys. Rev. A* **69**, 030301(R) (2004).

[13] You, J. Q., Hu, X. & Nori, F. Correlation-induced suppression of decoherence in capacitively coupled Cooper-pair boxes. *Phys. Rev. B* **72**, 144529 (2005).

[14] Shaw, M. D., Schneiderman, J. F., Palmer, B., Delsing, P. & Echternach, P. M. Experimental realization of a differential charge qubit. *IEEE Trans. Appl. Supercond.* **17**, 109-112 (2007).

[15] Medford, J., Beil, J., Taylor, J. M., Rashba, E. I., Lu, H., Gossard, A. C. & Marcus, C. M. Quantum-dot-based resonant exchange qubit. *Phys. Rev. Lett.* **111**, 050501 (2013).

[16] Taylor, J. M., Srinivasa, V. & Medford, J. Electrically protected resonant exchange qubits in triple quantum dots. *Phys. Rev. Lett.* **111**, 050502 (2013).

[17] Ando, T., Fowler, A. B. & Stern, F. Electonic properties of two-dimensional systems. *Rev. Mod. Phys.*, **54**, 437-672 (1982).

[18] Kelly, J., Barends, R., Campbell, B., Chen, Y., Chen, Z., Chiaro, B., Dunsworth, A., Fowler, A. G., Hoi, I.-C., Jeffrey, E., Megrant, A., Mutus, J., Neill, C., O'Malley, J. J., Quintana, C., Roushan, P., Sank, D.,Vainsencher, A., Wenner, J., White, T. C., Cleland, A. N. & Martinis, J. M. Optimal quantum control using randomized benchmarking. *Phys. Rev. Lett.* **112**, 240504 (2014).

[19] Ghosh, J., Coppersmith, S. N. & Friesen, M. Pulse sequences for suppressing leakage in single-qubit gate operations. Preprint at https://arxiv.org/abs/1612.00568 (2016).
